# Knowledge Is Power for Medical Assistants: Crystallized and Fluid Intelligence As Predictors of Vocational Knowledge

**DOI:** 10.3389/fpsyg.2018.00028

**Published:** 2018-02-01

**Authors:** Anne Moehring, Ulrich Schroeders, Oliver Wilhelm

**Affiliations:** ^1^Institute of Social Medicine and Prevention, University Hospital of Greifswald, Greifswald, Germany; ^2^Department Psychological Assessment, Institute of Psychology, University of Kassel, Kassel, Germany; ^3^Department Individual Differences and Psychological Assessment, Institute of Psychology and Education, Ulm University, Ulm, Germany

**Keywords:** knowledge-is-power, medical assistants, vocational education and training, crystallized intelligence, fluid intelligence

## Abstract

Medical education research has focused almost entirely on the education of future physicians. In comparison, findings on other health-related occupations, such as medical assistants, are scarce. With the current study, we wanted to examine the knowledge-is-power hypothesis in a real life educational setting and add to the sparse literature on medical assistants. Acquisition of vocational knowledge in vocational education and training (VET) was examined for medical assistant students (*n* = 448). Differences in domain-specific vocational knowledge were predicted by crystallized and fluid intelligence in the course of VET. A multiple matrix design with 3 year-specific booklets was used for the vocational knowledge tests of the medical assistants. The unique and joint contributions of the predictors were investigated with structural equation modeling. Crystallized intelligence emerged as the strongest predictor of vocational knowledge at every stage of VET, while fluid intelligence only showed weak effects. The present results support the knowledge-is-power hypothesis, even in a broad and more naturalistic setting. This emphasizes the relevance of general knowledge for occupations, such as medical assistants, which are more focused on learning hands-on skills than the acquisition of academic knowledge.

## Introduction

Research regarding medical education has mainly focused on medical students in different phases of their education by examining admission to medical schools and universities (Lievens et al., 2016; Schripsema et al., 2017), performance in school (Schauber et al., 2015), and the transition from university education to practice (Schmidt and Rikers, 2007). While physicians are undoubtedly of great importance for the health system, other health-related occupations are often neglected in research on medical education. More specifically, medical assistants are an important liaison between patients and physicians. The specific duties of a medical assistant vary depending on the type and size of the healthcare facility, and its specialization. In Germany, medical assistants mostly work in medical practices as assistants of the respective physician. Their duties typically include the implementation of medical treatments and especially the maintenance and management of administrative work (for a comprehensive overview of tasks see Taché and Chapman, 2006; Taché and Hill-Sakurai, 2010). Unlike nurses, who in many countries are required to complete a college or university education, medical assistants in Germany are trained in vocational schools that focus on hands-on practice and administrative work. Successful graduation from vocational training programs, such as the medical assistant training, is a necessary prerequisite to start a professional career. Moreover, the specialized medical and health-related knowledge acquired during medical vocational education and training (VET) is important for later work performance (Hunter, 1983; Lievens and Patterson, 2011).

In Germany, VET is characterized by a combination of traditional education in vocational schools and training of hands-on skills in medical practice. The regular training period for medical assistants is 3 years with an interim exam after the second year and the final graduation exam at the end of the third year. The curriculum of medical assistants in the German federal state Baden-Wuerttemberg comprises (a) medical terminology, (b) assistance with diagnostic and therapeutic actions for various diseases, (c) attending patients during preventive treatments, (d) how to maintain patient confidentiality, (e) medical data protection, (f) legal regulations relevant for their profession, and (g) occupational safety and health (Ministry of Education and Cultural Affairs, Youth and Sports of Baden-Wuerttemberg, 2005). Early dropouts of VET are both a personal risk for VET students and a cost factor for organizations offering VET. Empirical evidence has shown students with low training satisfaction or a mismatch between vocational interests and work requirements are more likely to leave VET without graduating (Judge et al., 2001; Volodina et al., 2015). A more comprehensive understanding about factors contributing to successful graduation from VET might improve career counseling and, in the long term, reduce dropout rates.

Considering the relevance of VET success from the perspective of educational and labor market policy, empirical findings on individual differences in vocational education are surprisingly scarce. With the current study, we add to the research literature by analyzing the joint and unique effects of gf and gc in explaining individual differences in vocational knowledge of future medical assistants. The implemented research design allows us to estimate these effects at different phases of VET and to elaborate regarding the extent to which the knowledge-is-power hypothesis holds true in a real-life educational setting.

The predictive validity of cognitive abilities, such as intelligence, for school and vocational success is one of the best established findings in social sciences. Numerous studies suggest that fluid and crystallized intelligence are crucial in various academic and occupational settings (Ree and Earles, 1991; Schmidt and Hunter, 1998; Kuncel and Hezlett, 2007). Fluid intelligence (gf) is understood as a domain-general ability to reason and is strongly related with, or even synonymous with, working memory capacity (Kyllonen and Christal, 1990; Wilhelm et al., 2013). Moreover, gf is often regarded as essential and prototypical for general cognitive ability (e.g., Marshalek et al., 1983), which is a powerful predictor of college and university success as well as job performance (Salgado et al., 2003; Ones et al., 2004; Kuncel and Hezlett, 2007). For health-related education, Reeve and Basalik (2014) found a substantial impact of cognitive ability as assessed with traditional gf tests for the prediction of several outcomes related to health literacy (i.e., “the ability to obtain, process, and understand basic information and services need to make appropriate health decisions,” p. 94), but no significant increment of specific health-related knowledge over and above gf. However, the significance of gf has rarely been investigated in VET settings. Furthermore, performance on non-verbal gf or working-memory measures is often taken as a proxy and equated with intelligence (Laidra et al., 2007; Furnham and Monsen, 2009). Several studies showed that a common general factor – often called g - is pivotal for educational success (Reeve and Basalik, 2014; Tucker-Drob et al., 2014). For example, Carretta and Doub (1998) found that for participants in technical training for various career fields, general ability was a better predictor of training outcomes than prior vocational knowledge. Nonetheless, the authors also found prior vocational knowledge was an important predictor of acquired vocational knowledge.

Besides gf, crystallized intelligence (gc) is another prominent factor in consensual theories on the structure of intelligence (Horn and Cattell, 1966; Carroll, 1993; McGrew, 2009). Gc is defined as acculturated knowledge across a broad range of domains (Cattell, 1971; Schipolowski et al., 2014). While gc is conceived as domain-general, people usually acquire in-depth knowledge or expertise in only a few domains (Kanfer and Ackerman, 2004). This would suggest that individuals develop a specific profile of their declarative knowledge according to their learning experiences and interests. Whereas such learning opportunities seem rather homogeneous in regular school education (Schroeders et al., 2015), individuals pursue different educational paths afterward and presumably develop distinct knowledge structures with little overlap (Kanfer and Ackerman, 2004). In the framework of the Cattell-Horn-Carroll (CHC) theory, domain-specific knowledge is captured with a separate factor (gkn), which is defined as deeply specialized knowledge (or ‘expertise’) acquired on a specific subject that does not typically represent the knowledge of the individuals culture (see also McGrew, 2009). If these considerations are accurate, medical assistant students should acquire specialized vocational knowledge, which constantly increases throughout the specialized education and later working life, and diminishes with retirement (Ackerman, 1996). However, the process of vocational knowledge acquisition in one specific domain is unrelated or even negatively related to knowledge acquisition in another domain. Cattell (1963) described the relations among gf, gc, and academic achievement in the Investment Theory, stating that fluid abilities are invested in the development of crystallized abilities. The coupling between gf and gc should be especially strong in early in school, but decreasing during late childhood and adolescence. In a comprehensive review, Baumert et al. (2009) concluded that domain-specific knowledge contributes to the performance in educational settings over and above fluid intelligence and that its influence is becoming more important with ongoing education.

Such findings corroborate with the knowledge-is-power hypothesis stating that cognitive endeavors in a specific domain are best predicted by domain-specific knowledge rather than more general cognitive abilities such as fluid intelligence (Hambrick and Engle, 2002). Accordingly, more knowledgeable students are better at acquiring new knowledge than students with less prior knowledge (i.e., Matthew effect, see Schroeders et al., 2016). The underlying mechanisms include a more accurate retrieval, deeper integration, and faster processing of new knowledge within a given domain. Although the hypothesis has initially been formulated in a general way, it has been tested solely in highly specific contexts (e.g., Chase and Simon, 1973; Hambrick and Engle, 2002). For example, Hambrick (2003) showed that prior basketball knowledge facilitated the acquisition of new basketball knowledge, whereas the effect of gf on learning was negligible. However, compared to specific sport-related knowledge, vocational knowledge of medical assistants, as gathered through VET, is much more heterogeneous and detailed, including metacognition and social skills (e.g., organizing, empathetic respond to patients’ needs). Thus, as suggested in the power-is-knowledge hypothesis, it is possible that gc better predicts vocational knowledge acquisition than gf in a broad educational, real-life setting. This raises the question: To what extent can the knowledge-is-power hypothesis be generalized to a more complex real-life setting? Specifically, we want to examine the influence of a broad gc-factor in line with Cattell’s (1971) definition of gc on vocational knowledge of medical assistant students.

Inconsistent findings reported in the research literature regarding the respective influence of gf or g and gc can be linked to several factors. First, the specificity of the learning situation, that is to say, gf is more important for learning outcomes in restricted environments (i.e., laboratory multimedia presentation), whereas it is less important for self-directed learning at home (Beier and Ackerman, 2005). In the context of VET, students usually attend lessons at school and apply the knowledge in the practical part of their education. Thus, knowledge is imparted in well-structured classes as well as in unregulated learning environments. Second, the relative contribution of gf and gc also depends on the learning outcome. Since the current study focuses on the scholastic aspects of VET, we expect a stronger influence of gc. Vocational knowledge is defined as “the accumulation of facts, principles, concepts, and other pieces of information that are considered important in the performance of one’s job” (Dye et al., 1993, p. 153). This definition emphasizes the relevance of gc compared to gf as a predictor of vocational knowledge acquisition. Third, the subject of learning, that is, the specific knowledge domain might be important for the influence of gf and gc on knowledge acquisition. Gf has been shown to have a stronger impact on learning in the physical sciences, such as physics and chemistry, whereas gc was a better predictor for knowledge in arts, humanities, and civics (Ackerman, 2000). With respect to health-related knowledge, such as nutrition, mental health, and illness, there is also a substantial predominance of gc compared to gf (Beier and Ackerman, 2003), which also points to a stronger effect of gc in the education of medical assistants.

In summary, the contributions of gf and gc to vocational knowledge depend on several factors. For the present study, we expected a positive influence of both gf and gc on vocational knowledge for medical assistant VET students. In accordance with the knowledge-is-power hypothesis, we expected gc to show a greater impact on vocational knowledge than gf. As an alternative way of modeling, individual differences in vocational knowledge acquisition during VET of medical assistants might also be explained by a general intelligence factor (g) (Schroeders et al., 2013) that accounts for the shared variance in gf and gc. Thus, we will present a model with a g-factor and a nested gc^#^ factor (i.e., residual knowledge factor) as predictor variables and compare results to the gf-gc-model. If the knowledge-is-power hypothesis holds true, gc^#^ should still have a stronger impact on vocational knowledge than g. Even though the study was cross-sectional, the research design allowed for an evaluation of vocational knowledge acquisition at the different stages of VET, thus, providing a rough estimation of learning trajectories. In terms of mean changes, we expect strong changes in VET knowledge as the education progresses, whereas we predict smaller changes in gc and no changes in gf.

## Materials and Methods

### Participants

A sample of *n* = 448 medical assistant students (438 female) was recruited from five out of 24 vocational schools that provide VET for medical assistants in the German federal state of Baden-Wuerttemberg. The high proportion of female students is similar to the overall percentage of female medical assistant students in Germany (98%; see Federal Statistical Office, 2016). Age of the participants ranged from 15 to 44 years (*M* = 20.02, *SD* = 3.45). Cohorts of VET students were recruited from all 3 years of education. Detailed information on the demographics is provided in **Table 1**. Medical VET students attended courses with the same curriculum irrespective of their previous education. Informed consent was obtained from all participants and individual feedback was offered to all participants. The students were tested in groups of 10–30 people, depending on the class size, who worked on the whole test battery for approximately 100 min.

**Table 1:** Sample characteristics.

	1st year	2nd year	3rd year
Sample size	172	127	149
Age *M* (*SD*)	19.0 (3.4)	20.5 (4.0)	20.7 (2.6)
Female [%]	99.4	99.2	98.6
School graduation [%]			
Vocational track (*Hauptschule*)	16.8	11.8	13.9
Intermediate track (*Realschule*)	72.5	71.8	77.2
Academic track (*Gymnasium*)	9.1	12.9	5.0
Others (e.g., mixed track schools)	1.5	3.5	4.0

### Measures

#### Domain Specific Knowledge Tests

Vocational knowledge of medical assistant students was assessed with a domain-specific knowledge test covering the different relevant topics of the VET course. The following sources were used for item development: (1) the curriculum of medical assistants (Ministry of Education and Cultural Affairs, Youth and Sports of Baden-Wuerttemberg, 2005), (2) standardized final and interim exams that were provided by the *Chamber of Industry and Commerce* [Industrie- und Handelskammer (IHK)] from the years 2010–2014 and official sample questions for exam preparation (e.g., Zimmermann, 2014), and (3) text books used in VET courses (e.g., Fox et al., 2008). Items had a multiple choice format with three distractors and one correct answer and covered the content of all 3 years of VET as described in the framework curriculum of medical assistants. In more detail, this includes: (1) medical knowledge that is relevant in patient care before, during, and after medical treatment; (2) laboratory knowledge, such as knowledge about hygienic standards or medical instruments as well as hands-on skills to analyze laboratory samples; and (3) knowledge about organizational aspects of a medical workplace and social interaction with patients which comprises the procurement and management of materials, accounting, documentation, and scheduling. A total of 118 items were tested in a pilot study with a sample of 292 medical assistant students in March and April 2014 to select 50 items for the final knowledge test, based on their difficulty (≥0.25 and ≤0.95) and part-whole corrected item-total correlation for each subscale (*r_bis_* ≥ 0.25). To keep the individual workload to a minimum, a multiple matrix design with three training-year specific booklets was used. More specifically, each test consisted of 30 items with 10 items being equal in all booklets, and another 10 items shared between adjacent test forms (see **Table 2**). We used a vertical linking design in order to connect the different training-year specific test forms and to estimate students’ abilities on a common scale (Kolen and Brennan, 2004). Further, a comparison between the three-dimensional factor model, based on the content domains, and a one-dimensional factor model with a χ^2^ difference test revealed a significant advantage of the three-dimensional model, Δχ^2^(292) = 8.17, *p* = 0.04. Therefore, medical knowledge, laboratory knowledge, and organizational knowledge were used as subtests for the domain-specific knowledge test.

**Table 2:** Vertical linking design across 3 years of vocational education and training.

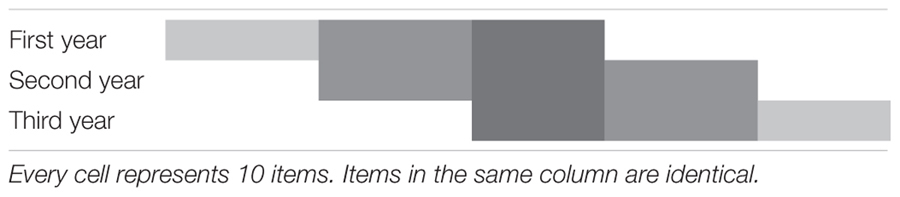

#### Fluid and Crystallized Intelligence

Both gf and gc were assessed with the *Berlin Test of Fluid and Crystallized Intelligence for Grades 8–10* (Wilhelm et al., 2014). Figural gf is considered prototypical for gf and was accordingly measured with the figural gf scale (Wilhelm, 2004). A sequence of geometric figures was presented and participants had to identify which were the next two figures in the sequence out of three alternatives for each missing figure. Participants worked on 16 items for a maximum of 14 min.

Gc was assessed in three broad domains: (1) natural sciences (e.g., Which of the following is the formula for hydrochloric acid?^1^ (a) HF, (b) HBR, (c) *HCI*, (d) HI), (2) humanities (e.g., Who was Friedrich Nietzsche? (a) a historian, (b) *a philosopher*, (c) a mathematician, (d) a chemist), and (3) social studies (e.g., What is the definition of gross national product (GNP)? GNP is a... (a) measure of the tax revenue of the government, (b) measure of the states’ social expenditure, (c) *measure of the income of residents in a national economy*, (d) measure of the export volume of a national economy). The gc test consists of 64 items covering the “breadth and depth” of the declarative knowledge (Horn and Noll, 1997, p. 69). Test time was 20 min.

### Data Analysis

First, we established measurement models for gf and gc in the framework of *Confirmatory Factor Analysis* (CFA) with the *Weighted Least Squares Mean and Variance adjusted* (WLSMV) estimator which is appropriate for dichotomous variables (Beauducel and Herzberg, 2006). According to Yu (2002, pp 94–95) the following cut-off values indicate good model fit: *Comparative Fit Index* (CFI) ≥ 0.95, *Root Mean Square Error of Approximation* (RMSEA) ≤ 0.06, and *Weighted Root Mean Square Residual* (WRMR) ≤ 1.0. In subsequent analysis, items of the intelligence measures were parceled to keep the number of indicators within a reasonable range and to get reliable and robust parameter estimates. The parceling approach is viable in the case that the construct is unidimensional and the residual correlations are negligible (Little et al., 2002). For figural gf, five parcels with almost similar average difficulty were compiled. For gc, the items were parceled according to the three broad content domains of science, humanities, and social studies. Due to the vertical linking design (Kolen and Brennan, 2004) of the domain-specific knowledge tests, two-parameter logistic (2PL) models, which are equivalent to CFA models with the WLSMV estimator (Asparouhov and Muthén, 2015), were estimated for each year. The resulting *Weighted Likelihood Estimates* (WLEs; Warm, 1989) for the three domains (medical knowledge, laboratory knowledge, and organizational knowledge) represent students’ domain-specific knowledge after linking items on a common scale according to Haberman (2009). Scaling and linking within in an IRT (item response theory) framework were conducted with the R packages *TAM* (Kiefer et al., 2016) and *sirt* (Robitzsch, 2016).

In order to make valid comparisons of students’ performance across years of VET, it is necessary to ensure measurement invariance for gf and gc. Invariance testing with *Multiple Group Confirmatory Factor Analysis* (MGCFA; Cheung and Rensvold, 2002) is a sequential and straightforward procedure of constraining more and more measurement parameters (factor loadings, intercepts, and residual variances) to be equal across groups (i.e., years of VET education). Different levels of invariance are assessed by comparing measurement models, from the least to the most restrictive model. First, *configural invariance* is tested by freely estimating all measurement parameters while all factor means are fixed to zero for identification purpose. For *metric invariance*, factor loadings are additionally fixed to equality between groups. In the next step, *scalar invariance*, the intercepts are also fixed to equality, but means were freely estimated in all except one group. Finally, to test *strict invariance*, residual variances were fixed to equality between groups. While metric invariance is sufficient to compare the bivariate relations (i.e., regressions and correlations) between latent variables across groups, scalar invariance is necessary for the comparison of the mean structure.

## Results

### Measurement Models

One-dimensional measurement models were estimated for gf and gc with individual items as indicators. For the gf scale, one item had to be deleted due to a technical problem. The measurement model with 15 gf items showed good fit to the data: χ^2^(77) = 93.5, *p* = 0.09, CFI = 0.982, RMSEA = 0.02, WRMR = 0.854. For gf, five parcels with equal difficulty were built. For the gc scale, the four medicine items were excluded from all further analysis due to a substantial overlap with the curriculum of medical assistants. Although the CFI for the gc model was slightly below the cut-off value suggested by Yu (2002), other fit indices indicated good model fit: χ^2^(1,539) = 1,725.8, *p* < 0.01, CFI = 0.942, RMSEA = 0.02, WRMR = 0.993. For subsequent analysis indicators were parceled in order to reduce the number of estimated parameters and to allow for robust parameter estimation with moderate sample size. Gc items were aggregated to parcels according to three broad content areas. To ensure the comparability of the intelligence scales across the 3 years of VET, the measurement models were tested for measurement invariance. The models were compared based on the deterioration in the CFI between consecutive models with ΔCFI ≤ 0.01 indicating invariance (Cheung and Rensvold, 2002). For all measurement models, strict measurement invariance was given (see **Table 3**), which allows us to examine the relative contribution of gf and gc on vocational knowledge across years of education.

**Table 3:** Measurement invariance testing for Gf and Gc.

	χ^2^/*df*	*p*	RMSEA	CFI	ΔRMSEA	ΔCFI
**gf**						
Configural	14.2/15	0.51	<0.001	1.0	–	–
Metric	27.1/25	0.35	0.02	0.994	–0.02	0.010
Scalar	35.5/33	0.35	0.02	0.992	0.00	–0.002
Strict	40.9/43	0.56	<0.001	1.0	0.02	–0.008
**gc**						
Configural	–	–	–	–	–	–
Metric	8.7/4	0.07	0.08	0.992	–	–
Scalar	16.9/8	0.03	0.08	0.985	0.00	0.007
Strict	20.7/14	0.11	0.06	0.988	0.02	–0.003

*gf, fluid Intelligence; gc, crystallized Intelligence. CFI, Comparative Fit Index; RMSEA, root mean square error of approximation.*

For the vocational knowledge test, three-dimensional 2PL models were estimated separately for each year of VET and subsequently linked on a common scale (Haberman, 2009). Five of the original 50 items (1 item from medical knowledge, 2 items from laboratory knowledge, and 2 items from organizational knowledge) had to be excluded from the vocational knowledge test due to negative discrimination parameters, resulting in booklets of 27 items for the first and second year each, and 29 items for the third year. Person estimates for each content domain of the vocational knowledge test were used as indicators in the subsequent SEM.

**FIGURE 1:**
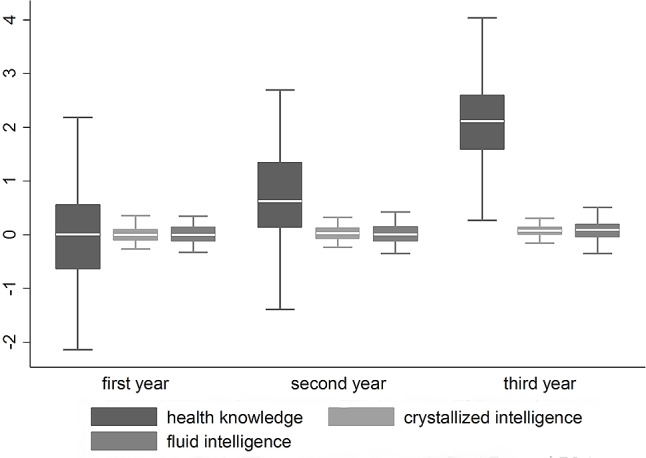
Factor means of health-related vocational knowledge, crystallized, and fluid intelligence. Parameters are means of the latent variables from the measurement models of health knowledge, crystallized, and fluid intelligence on the level of strict invariance. Boxes represent the interquartile range; whiskers represent the standard deviations.

There were significant increases in vocational knowledge, which indicates the successful acquisition of job-relevant knowledge over the course of VET. The standardized mean differences, according to Cohen (1988), ranged from moderate to high effects (*d*_1/2_ = 0.45; *d*_2/3_ = 0.99; *d*_1/3_ = 1.51). In contrast, the means of gf and gc remained stable across the 3 years of education (see **Figure 1**), since domain-general knowledge and fluid intelligence are not specifically promoted through VET. The almost identical means of the intelligence scales across VET in this cross-sectional data, also advocate for the comparability of the subsamples.

### Prediction of Domain Knowledge in Health-Related VET

**FIGURE 2:**
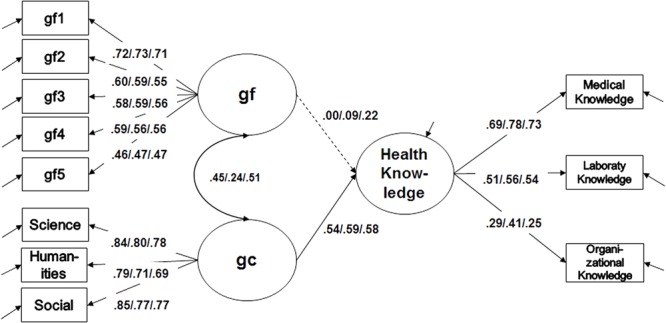
Prediction model of vocational knowledge through gf and gc. Fluid (*gf*) and crystallized (*gc*) intelligence as correlated factors on the level of scalar invariance; non-significant parameters are represented with a dashed line. *n* = 448, χ^2^(155) = 212.3, *p* < 0.01, CFI = 0.952, RMSEA = 0.05, SRMR = 0.07.

Structural equation modeling was used to predict individual differences in health knowledge of medical assistants with gc and gf (**Figure 2**). Constraining the factor loadings and intercepts to equality across years of education (i.e., strong measurement invariance), the model still provided good model fit [χ^2^(155) = 212.3, *p* < 0.01, CFI = 0.952, RMSEA = 0.05, SRMR = 0.07] as compared to a model constrained to metric measurement invariance (ΔRMSEA = 0.00, ΔCFI = 0.007). In the correlated factor model, gc turned out to be the strongest predictor of vocational knowledge in all 3 years of VET. Interestingly, in this model, gf showed no impact on vocational knowledge for the first 2 years of education and only a small influence in the last year of VET.

**FIGURE 3:**
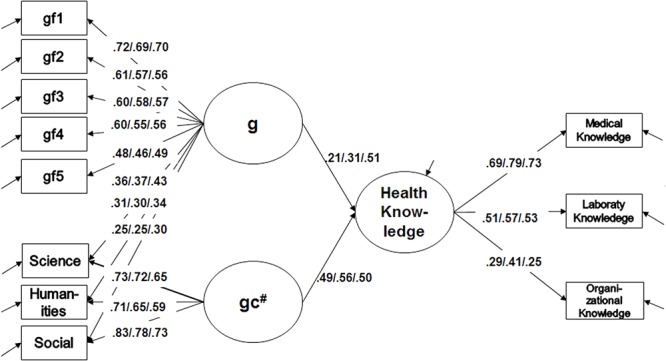
Prediction model of vocational knowledge through g and gc^#^. Crystallized intelligence nested (*gc^#^*) below intelligence (*g*) on the level of scalar invariance. *n* = 448, χ^2^(155) = 207.9, *p* < 0.01, CFI = 0.956, RMSEA = 0.04, SRMR = 0.07.

To further examine the unique effects of gf and gc on vocational knowledge acquisition, we modeled gc as a nested factor (labeled gc^#^) below an overarching g factor (see **Figure 3**). This allows us to estimate the effect of a general cognitive ability factor and an independent (residual) knowledge factor. Model fit of the nested factor model was good: χ^2^(155) = 207.9, *p* < 0.01, CFI = 0.956, RMSEA = 0.04, SRMR = 0.07. As for the nested factor model, the overarching g factor showed a significant influence on health knowledge with a slight increase for the third year students. However, the predictive power for gc^#^ was higher than the impact of g, with the exception of the third year. And even for third year students, the influence of an overarching g factor did not exceed that of gc^#^.

## Discussion

While most studies in medical education research are limited to university education of prospective physicians, the current study focused on knowledge acquisition and prediction in VET of medical assistants. More specifically, individual differences in domain-specific knowledge at different stages of VET were regressed on gf and gc. One of the main finding is that gc turned out to be the strongest predictor of domain-specific knowledge throughout the course of education, emphasizing the importance of general knowledge for educational achievements. It is important to keep in mind that the implemented gc test covered knowledge in various domains, such as technology, music, and law, rather than health-related or medical knowledge. This result is in line with previous findings emphasizing the importance of prior knowledge in learning. For example, Hambrick and colleagues showed that prior baseball knowledge was the most important predictor for baseball-related memory performance (Hambrick and Engle, 2002), current events knowledge (Hambrick et al., 2007), and knowledge acquisition (Hambrick, 2003). Compared to those previous investigations of the knowledge-is-power hypothesis, the present study offers a broad assessment of knowledge acquisition in VET. Thus, the breadth and depth of the learning subject differs from laboratory studies about narrow domains such as baseball knowledge. Furthermore, we assessed general knowledge instead of prior domain-specific knowledge as a predictor of successful knowledge acquisition, which allows for an assessment of gc that is in line with Cattell’s (1963) definition as a result of invested intelligence.

The respective contributions of gf and gc are influenced by several factors: Most prominently, a stronger influence of gf has been reported on knowledge acquisition in classroom settings as compared to self-study sessions (Beier and Ackerman, 2005). In the context of knowledge acquisition in VET, the classroom setting represents a formalized learning environment, which is accompanied by additional learning settings in a medical practice. The education in school and in medical practice is approximately split in a ratio of 40–60%, which emphasizes the importance of vocational knowledge in practical VET settings. Since these settings are less regulated than secondary education with full-time class attendance, prior knowledge is crucial for the VET students’ learning success. Thus, higher levels of gc allow students to more easily integrate new knowledge into a framework of prior knowledge in new and less regulated situations. This unique learning environment might explain the small influence of gf on vocational knowledge acquisition of medical assistants. Considering the dominance of the assessment of gf in explaining and predicting learning outcomes (e.g., Salgado et al., 2003; Kuncel et al., 2004; Ones et al., 2004; van der Maas et al., 2006) this result is remarkable. The way gf was measured in the current study might have biased the results. More specifically, although figural gf is the best proxy for the assessment of gf, it does not replace a comprehensive assessment of gf (Wilhelm, 2004). A broader assessment of gf, taking into account verbal and numerical gf, might enhance the effect of gf. Alternatively, working memory could be used as a predictor of domain-specific knowledge acquisition (Hambrick and Engle, 2002).

The differential contributions of gf and gc should also be interpreted in light of the high construct overlap between both factors, which some researchers interpret as an indication of a general cognitive ability factor (e.g., Carroll, 1993). However, in the present study, the predictive power of a general g factor accounting for gf as well as the shared variance between gf and gc did not exceed the impact of the residual knowledge factor gc#, which was nested below an overarching g factor. Thus, even under the assumption that a general cognitive ability factor accounts for the high construct overlap between gf and gc, the importance of the knowledge factor for the prediction of vocational knowledge is clearly visible.

The predictive power of fluid intelligence slightly increased for third-year students, which might be due to the curriculum that continuously emphasizes independent planning of practice-oriented tasks (e.g., organizing and assisting in surgical treatments) and complex problem solving (e.g., recognizing suspicious laboratory values), rather than focusing on declarative knowledge (Ministry of Education and Cultural Affairs, Youth and Sports of Baden-Wuerttemberg, 2005). However, it is likely that these changes in the power of gf toward the third year are artifacts of random fluctuations due to the small sample sizes in each grade. As expected, we found a substantial increase of vocational knowledge between adjacent years of VET and no changes in gf and gc. The effect sizes of the increase in vocational knowledge in the present study are slightly higher than the typically moderate effects in secondary school education (Beaton et al., 1996; Schroeders et al., 2015). This supports the assumption that in-depth knowledge especially develops after regular school education, when students are able to choose more specific paths of education. This vocational, in-depth knowledge is primarily acquired in a few domains, resulting in distinct knowledge profiles (Kanfer and Ackerman, 2004).

The current findings provide support for the notion that a comprehensive knowledge base is a crucial prerequisite for knowledge acquisition in educational environments (Baumert et al., 2009, 2012). The results of Baumert et al. (2009) show an increasing influence of gc and a decreasing influence of gf on academic achievement from the end of elementary education to the upper secondary level. This is in line with the current findings, showing a strong impact of gc and a negligible effect of gf. The cumulative process of knowledge acquisition is favored by the hierarchically structured VET curriculum. Students with higher levels of gc may generally be able to acquire new knowledge more quickly and efficiently. Thus, newly acquired knowledge is integrated in a framework of prior knowledge. According to Cattell’s theory of fluid and crystallized intelligence (Cattell, 1943, 1963), aside from gf, factors such as motivation and teaching are essential for learning. In other words, while fluid intelligence is necessary for learning, it is not sufficient. It seems likely that students who are more interested in the subject and more motivated to succeed in VET, perform better in vocational knowledge tests. Students need the opportunity and the motivation to invest their intelligence constantly over a long period of time to study relevant material. Therefore, knowledge tests can also be understood as indirect measures of motivation. This has been pointed out by several studies, for example, in the prediction of vocational knowledge of pilot applicants (Zierke, 2014). The strong influence of gc was interpreted as an indication that the educational success includes both ability in a sense of what the student “can do” as well as motivation as the “will do” portion of educational achievement (King et al., 2013), which can effectively be assessed with knowledge tests.

The current results support the notion that knowledge tests might be a useful selection tool in the context of career counseling of high school students. While non-cognitive constructs such as vocational interests contribute to dropout intention, job change, or job satisfaction (e.g., Barrick and Mount, 1991; Barrick et al., 2001; Verquer et al., 2003; Volodina et al., 2015), the actual academic performance is still best predicted by measures of cognitive ability. Although career counseling is often strongly focused on motivational aspects, tests of maximum performance such as power tests are more resistant to faking and have high predictive validity for vocational knowledge acquisition in VET.

## Ethics Statement

All subjects gave written informed consent. The protocol was approved by the “Ministerium für Kultus, Jugend und Sport Baden-Württemberg.”

## Author Contributions

OW and US conceived and designed the study. AM performed data collection, pre-processing and analysis of the data. US helped with the data analysis. AM wrote and OW and US critically commented on the manuscript. AM, US, and OW gave final approval of the manuscript to be published.

## Conflict of Interest Statement

The authors declare that the research was conducted in the absence of any commercial or financial relationships that could be construed as a potential conflict of interest.

## Footnotes

1 Correct answer in italics.
